# Shower thoughts: why scientists should spend more time in the rain

**DOI:** 10.1093/biosci/biad044

**Published:** 2023-06-07

**Authors:** John T Van Stan, Scott T Allen, Douglas P Aubrey, Z Carter Berry, Matthew Biddick, Miriam A M J Coenders-Gerrits, Paolo Giordani, Sybil G Gotsch, Ethan D Gutmann, Yakov Kuzyakov, Donát Magyar, Valentina S A Mella, Kevin E Mueller, Alexandra G Ponette-González, Philipp Porada, Carla E Rosenfeld, Jack Simmons, Kandikere R Sridhar, Aron Stubbins, Travis Swanson

**Affiliations:** Department of Biological, Geological, and Environmental Sciences at Cleveland State University, Cleveland, Ohio, United States; Department of Natural Resources and Environmental Science at the University of Nevada-Reno, Reno, Nevada, United States; Savannah River Ecology Lab and with the Warnell School of Forestry at the University of Georgia, Athens, Georgia, United States; Department of Biology at Wake Forest University, Winston-Salem, North Carolina, United States; Terrestrial Ecology Research Group at the Technical University of Munich, Freising, Germany; Department of Water Management at the Delft University of Technology, Delft, the Netherlands; Dipartimento di Farmacia at the University of Genoa, Genoa, Italy; Department of Forestry and Natural Resources at the University of Kentucky, Lexington, Kentucky, United States; Research Applications Laboratory, at the National Center for Atmospheric Research, Boulder, Colorado, United States; Department of Soil Science of Temperate Systems, Agricultural Soil Science, at Georg-August-Universität, Göttingen, Germany; Peoples Friendship University of Russia, Moscow, Russia; National Public Health Center, Budapest, Hungary; Sydney School of Veterinary Science, at the University of Sydney, Sydney, New South Wales, Australia; Department of Biological, Geological, and Environmental Sciences at Cleveland State University, Cleveland, Ohio, United States; Department of City and Metropolitan Planning and with the Natural History Museum of Utah at the University of Utah, Salt Lake City, Utah, United States; Department of Biology at Universität Hamburg, Hamburg, Germany; Department of Minerals and Earth Sciences at the Carnegie Museum of Natural History, Pittsburgh, Pennsylvania, United States; Department of Philosophy and Religious Studies at Georgia Southern University, Statesboro, Georgia, United States; Department of Biosciences at Mangalore University, Konaje, Mangaluru, Karnataka, India; Departments of Marine and Environmental Science, Civil and Environmental Engineering, and Chemistry and Chemical Biology at Northeastern University, Boston, Massachusetts, United States; Independent Scholar Savannah, Georgia, United States

**Keywords:** extreme event biogeochemistry, field and laboratory studies, sampling bias, climate change, precipitation, condensation, ecosystem functioning

## Abstract

Stormwater is a vital resource and dynamic driver of terrestrial ecosystem processes. However, processes controlling interactions during and shortly after storms are often poorly seen and poorly sensed when direct observations are substituted with technological ones. We discuss how human observations complement technological ones and the benefits of scientists spending more time in the storm. Human observation can reveal ephemeral storm-related phenomena such as biogeochemical hot moments, organismal responses, and sedimentary processes that can then be explored in greater resolution using sensors and virtual experiments. Storm-related phenomena trigger lasting, oversized impacts on hydrologic and biogeochemical processes, organismal traits or functions, and ecosystem services at all scales. We provide examples of phenomena in forests, across disciplines and scales, that have been overlooked in past research to inspire mindful, holistic observation of ecosystems during storms. We conclude that technological observations alone are insufficient to trace the process complexity and unpredictability of fleeting biogeochemical or ecological events without the shower thoughts produced by scientists’ human sensory and cognitive systems during storms.

When caught in the rain, we have all run for cover—often to a nearby tree. On the way, we step over ephemeral puddles and hastily formed streams, marveling at how quickly the soil changes from supportive and predictable to untrustworthy: slippery, soft, and spongy. Waiting out the storm, we may move to avoid the increasingly drippy areas overhead, eventually leaning on the trunk to rest. Then, as the canopy saturates, water flows down the bark in rivulets, soaking our backs. Perhaps we escape at first chance, forgoing further observation. However, for natural scientists (researchers, educators and students), these experiences can reveal ephemeral phenomena, prompting curiosity and novel insights.

Human observation during storms has profoundly affected our understanding of ecosystems, from the earliest recorded botanical observations (Theophrastus’ *Historia Plantarum*) and indigenous practices. For example, the Bimbache community of El Hierro (Canary Islands) observed water running down tree bark during fog events and captured it for drinking, washing, and agriculture (Galindo and Glass 1764). If more contemporary hydrologists had watched the raking of fog by trees, forest managers may not have logged the Bull Run watershed (Portland, Oregon, in the United States), which reduced local precipitation inputs by 30% (Ham 1982). Given that direct observations are often infeasible, remote observation systems are crucial for capturing phenomena that are frequent, long lasting, or not easily predicted, but this introduces limitations to what we perceive. An unintended consequence of their deployment is that many scientists may not enter the storm, instead forming perceptions on the basis of sensor data while staying dry. Consequently, their scope of inference and ponderings may omit the impacts, flows, and drips that transport water through ecosystems during storms. What stormy phenomena remain unknown or are overlooked or misunderstood because of our absence in ecosystems during foggy, rainy, or snowy periods? Could our dry and technological biases limit the progress of natural science (Chu and Evans 2021) by constraining the “what if” and “I wonder how” musings that often inspire research and enrich environmental education (Futuyma 1998, Dangles and Casas 2012)?

Water science faces criticism regarding its alleged conceptual and theoretical stagnation (Nature Sustainability 2021) because of a “techno optimism that tries to solve all problems despite not asking fundamental questions” (Scarrow 2021). We argue that this issue extends beyond water science, because modern natural scientists often approach their study systems beneath an umbrella perspective—a limited viewpoint that obscures phenomena occurring just before, during, and after storms. Consistent with this thesis, philosopher Martin Heidegger argued that “Modern technology *is not applied to* natural science, far more [often] is modern natural science *the application of the essence of technology*” (Heidegger 1977). Therefore, although remote sensing and virtual experimentation with models are useful, their utility is limited because they cannot measure or test the phenomena or hypotheses that we have not yet observed or imagined. Mitigating these blind spots through mindful observations throughout storms may yield various benefits, including improved leveraging of technological sensing, sampling, and models. Real-time observation of storm-related phenomena could shine light on processes currently shadowed beneath umbrella perspectives. Indeed, many scientific breakthroughs were not products of technological advancement itself but were enabled by using new technology as an extension of the human observation system (e.g., Antoine-Laurent Lavoisier's early hydrogeological research; Meldrum 1933, Rappaport 1967) and imagination (e.g., eddy covariance systems permit verification of theoretical estimates of momentum, heat, and gas exchanges from ecosystems; Foken et al. 2012).

Humans are sophisticated sensor systems with high-frequency sound, sight, and smell detection, integrated with distributed temperature and pressure sensing across our bodies, etc. However, we have many limitations (e.g., being relativistic, uncalibrated, or state-dependent or having low recording capacity and biased memory). Technology counters these limitations but is most effective when complemented by human input. Human experience in the storm builds our intuition—motivating the expansion of technology's observational capabilities. Finally, the shower thoughts of scientists integrate technological observations, model hypotheses, and field realities into general theory for further testing.

In the present article, we present examples across disciplines, focused on forests (table 1, figure 1), as evidence of the need for natural scientists to emerge from beneath the umbrella and get wet. Accelerating climatic changes and the number of extreme events add urgency and opportunity to this cause. The amount, frequency, and intensity of precipitation are increasing in many parts of the world (Masson-Delmotte et al. 2021); therefore, whatever happens when it is wet and stormy, more of it is coming to a forest not so far from you. Scientists are increasingly attentive to how such precipitation shifts affect ecosystems, but there are still signs of a dry bias (Hubbart et al. 2016, Asbjornsen et al. 2018, Esteban et al. 2021). This bias likely varies geographically and by discipline, seeming less evident in research inherently tied to wet conditions (e.g., amphibian studies; Walls et al. 2013, Lowe et al. 2019) and regional- to global-scale studies using remote sensing and modeling methods (e.g., He et al. 2020) than in smaller-scale studies (i.e., the scale of human experience). For example, recent years have birthed a plethora of research focused on how drought affects plant–environment interactions at the scale of individual plants or small plant communities, possibly because drought impacts are more visually apparent and convenient to observe. But global warming has approximately equal impact on dry and wet extremes, and precipitation is increasing, on average, at the global scale (Masson-Delmotte et al. 2021), so it is problematic if fewer scientists are watching their systems more closely in and directly after storm events (as they do during and after drought; Hubbart et al. 2016) or experimenting with increased precipitation or simulated flooding (Person and Ruess 2003), compared with the seemingly ubiquitous rainout shelter (Asbjornsen et al. 2018).

**Figure 1.**
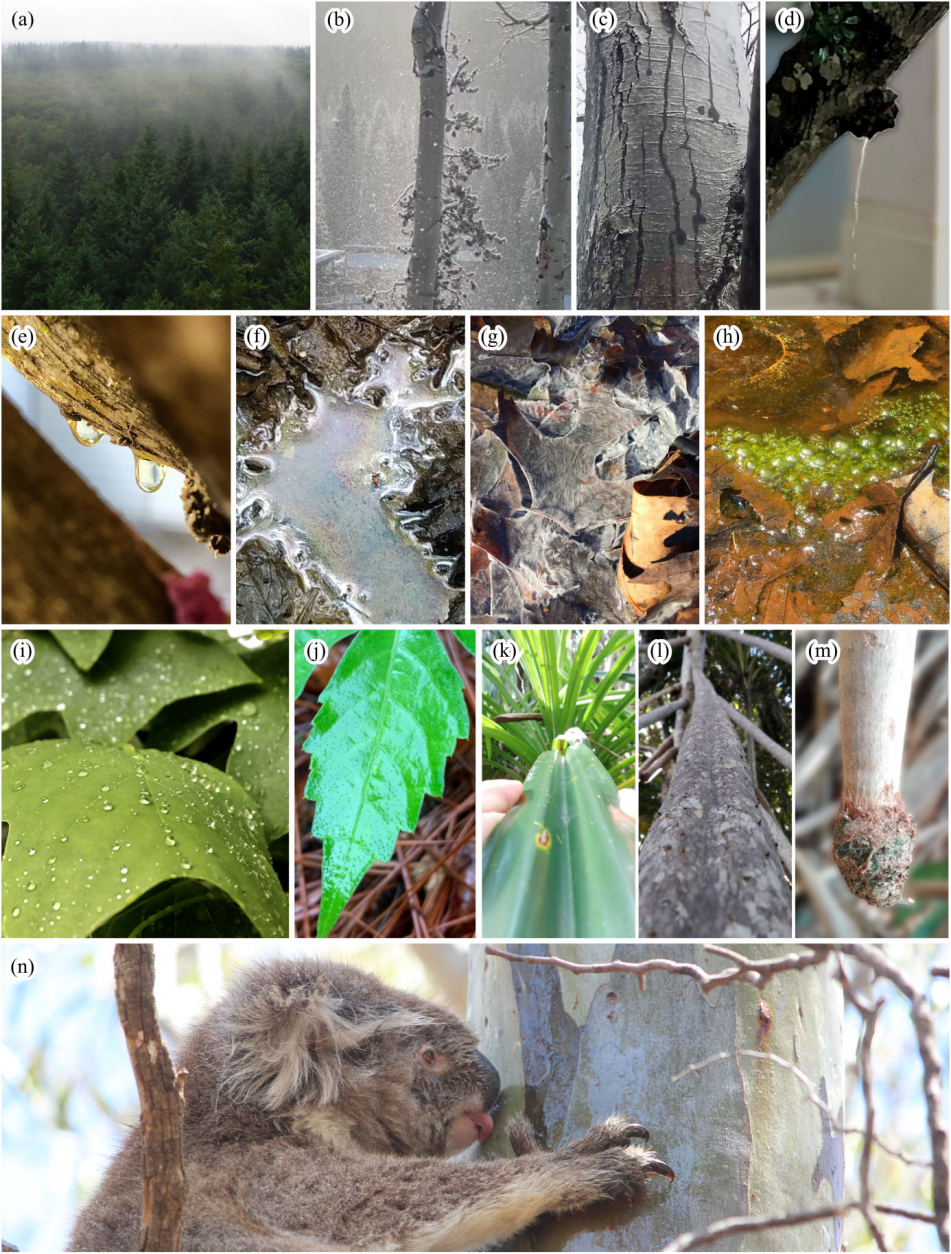
Photographs of example storm-related phenomena and indicators in forests observable to the human eye but difficult for remote technological systems to record. Plumes of (a) condensed vapor above a canopy and (b) wind-blown snow being redistributed. (c) Chemically enriched meltwaters can be seen draining down this trunk beneath the ice layer. (d) Drip point where rainfall is concentrated by the up-gradient canopy area. (e) Throughfall droplets gleaming amber, indicating light-absorbing dissolved organic matter. (f) Oil-like sheen produced by iron-oxidizing bacteria. (g) Streamers of elemental sulfur-containing bacteria (***Thiothrix*** sp.) in a small sulfide-rich spring. (h) Green chloroplasts of photosynthesizing cyanobacteria and algae. Leaf surface wetting patterns may range from (i) minimal coverage by small droplets to (j) full coverage by a thin film. ***Pandanus forsteri***’s (k) trough-like leaves and (l) branches that direct rainfall to (m) aerial root tips. (n) Koala drinks stemflow. Photographs: (a) AMJC-G; (b) EDG; (c) image from video at https://imgur.com/hgemi5E; (d) JTVS; (e) JTVS; (f) KEM; (g) J. Cosmidis; (h) CER; (i) JTVS; (j) ZCB; (k–m) MB; (n) VSAM, Koala Clancy Foundation.

**Table 1.** Response of various forest ecosystem components to storms, focusing mainly on the responses that are difficult to observe with technological equipment.

Response of	Examples	References
Energy	Wind variability/turbulence	Ruchith and Ernest Raj (2020)
	Droplet impacts and scouring flows	Dunkerley (2020)
	Vapor plumes and trapped water vapor in understory	Jiménez-Rodríguez and colleagues (2021), Jiménez-Rodríguez and colleagues (2020)
	Rates of canopy snow sublimation versus melt	Lundquist and colleagues (2021), Levia and Underwood (2004)
Pools	Litter and soil organic matter	Qualls (2020)
	Dissolution of nutrients along bedrock–soil interface	Backnäs and colleagues (2012)
	Filling or overflow of canopy water impoundments (dendro- or phytotelmata)	Mendieta-Leiva and colleagues (2020)
	Organismal biomass in litter and soil	Ptatscheck and colleagues (2018)
Fluxes of matter	Water: Novel or preferential flow paths through canopy, over soils, through soils	Weathers and colleagues (2020), Herwitz (1986), Friesen (2020)
	Particles: Topsoil erosion and transport, washout of captured aerosols	Dunkerley (2020), Ponette-González and colleagues (2022)
	Solutes: Canopy to soil nutrient returns, pollutant input, allelochemicals	Parker (1983), Klučiarová and colleagues (2008), Molina and colleagues (1991)
	Gasses: Carbon dioxide birch effect, nitrous oxide flush, leaf gas exchange	Unger and colleagues (2010), Enanga and colleagues (2016), Berry and colleagues (2019)
Microorganisms	Resuscitation of dormant microorganisms	Placella and colleagues (2012)
	Cell lysis by osmotic pressure	Bottner and colleagues (1998)
	Dispersal of fungal spores, phyllosphere bacteria	Magyar and colleagues (2021), Teachey and colleagues (2018)
	Microsites where microbes switch to alternative terminal electron acceptors	Burgin and colleagues (2011), Keiluweit and colleagues (2016)
Vegetation	Dispersal and establishment of reproductive materials Washout of plant-generated materials, such as pollen and nectars Novel water transport and uptake systems Activation of nonvascular vegetation	Reski (2018), Barthlott and colleagues (2014) Verstraeten and colleagues (2019), Campbell and colleagues (2013) Biddick and colleagues (2018) Porada and colleagues (2023),
Animals	Larval development of mosquitos and other animals in or around tree holes	Fish and Carpenter (1982), Kirsch and colleagues (2021)
	Animal consumption of free water and excretions into water flows	Mella and colleagues (2020), de Albuquerque and colleagues (2021), Beard and colleagues (2002)
	Behaviors that directly engineer water processes in ecosystems	Maschwitz and Moog (2000)
	Trophic structure and interactions	Romero and colleagues (2020), Skagen and Adams (2012)
Signaling	Flush pathogens or stress indicators from phyllosphere	Van Stan and colleagues (2020)
	Flush of organismal or waste products from insect infestation	Arango and colleagues (2019)
	Flush of byproducts from canopy and epiphyte life events	Guidone and colleagues (2021)
	Geomorphological alteration (over multiple events)	Lipar and colleagues (2021)

Changing precipitation regimes interact with changing disturbance regimes, such that effects compound. In vegetated ecosystems, disturbances that become more severe with climate change, such as fire (Keeley et al. 2019) and infestation (Jactel et al. 2019), can alter the amount and quality of canopy surfaces, affecting canopy–storm interactions (e.g., the canopy's ability to moderate intense rainfall; Keim and Link 2018), which can lead to ecosystem consequences (e.g., increasing soil erosion; Dunkerley 2020). As these changes take place, it becomes increasingly important for researchers and students to actively observe and experience the dynamic processes occurring within these ecosystems during storms. This firsthand exposure to storm-related phenomena helps to build a more comprehensive understanding of the complex interplay between climate change, disturbances, and ecosystem responses. By engaging in direct observation, scientists and students can identify new patterns and relationships that may otherwise go unnoticed in a rapidly changing environment. Therefore, we suggest that natural scientists and students who study the impacts of climate change on ecosystems have a special need to get wet, literally and figuratively. Experiencing storm-related phenomena directly can enhance pedagogy by deepening students’ understanding, fostering curiosity, and strengthening their connection to nature. This hands-on approach enriches all levels of environmental education, inspires research, and prepares future scientists for impactful contributions.

## What's beyond our umbrella science? Examples from forests

### Ecohydrology

Our umbrella perspective has resulted in ecosystem scientists knowing little about the filling and emptying of water within forest components as it drains through the overstory, understory, litter, and soil, or evaporates to the atmosphere (Coenders-Gerrits et al. 2020). Reviews on rain–canopy and snow–canopy interactions show that many land surface models have severely limited observational bases for storage estimates (Lundquist et al. 2021), have substantial variability in process representation (Gutmann 2020), or are missing spatiotemporally concentrated fluxes between reservoirs, such as the water that drains down plant stems, stemflow (Murray et al. 2013). Depending on the interactions between storm and canopy conditions, surfaces may be saturated in minutes, but this water could evaporate over the following hours (or days for snow). Land surface models, however, often compute canopy water and energy balances with a fixed time step that may be inconsistent with evaporation's actual timing. This can result in models predicting the canopy is dry when, in reality, it is wet (Llorens et al. 2014, Binks et al. 2021). In addition, the seasonal precipitation timing, associated meteorological conditions, and type (rain, snow, mixed, etc.) can play significant roles in canopy water storage, retention, and redistribution to the surface, with significant down-gradient effects on rivers and water management (Berghuijs et al. 2014, McCrystall et al. 2021).

Solving such issues with technology is challenging. Sensors measuring humidity and water vapor flux over canopies may see less precisely during or may be blinded by precipitation (Allen et al. 2020, Coenders-Gerrits et al. 2020)—resulting in blind spots regarding related cycles (such as carbon) measured by eddy covariance. Even when technology is properly monitoring areas of interest, moisture contributions from low-lying fog events (Izett et al. 2019), vapor trapped beneath the canopy (Schilperoort et al. 2020), or condensate plumes (figure 1a) may sneak into (or out of) the system, undetected by remote sensors. Catching these phenomena with human eyes could inform canopy water budgets and amelioration of leaf water deficits (Berry et al. 2019, Weathers et al. 2020). In cold regions or seasons, technological monitoring may miss snow redistributed from canopies to the surface via wind (figure 1b), sublimation (Drake et al. 2019), or meltwater drainage driven by a tree's low bark albedo or internal heat (figure 1c), affecting snow water storage estimates at scales relevant to forest and water management (Levia and Underwood 2004, Dickerson‐Lange et al. 2021). These issues result in land surface models using a wide variety of formulae and parameters for storm–vegetation interactions, indicating that we have a poor understanding of how to model these processes at large scales (Gutmann 2020). Therefore, direct observations from scientists regarding when and where unique ecohydrological conditions emerge could result in a synergy between human observation and technological advancement.

### Biogeochemistry and microbial ecology

Storms can rapidly soak ecosystems, accelerating the flushing, recharge, runoff, and transport of solids and solutes, reactivating interactions with microorganisms (McClain et al. 2003), acting as stirrers to force reactions outside of equilibrium or steady states. As climates change, stirring is changing too as storm frequencies or intensities increase in some regions (Pendergrass 2018, Tan et al. 2019) and decrease in others (Pokhrel et al. 2021) or vary in timing as seasonality changes (Konapala et al. 2020). All of these cases will have biogeochemical implications (Gutiérrez del Arroyo and Silver 2018, Deng et al. 2021). Predicting where and when hotspots and hot moments will arise in relation to storm events is, however, not straightforward.

Forests provide clues for human observers to infer where storm-related biogeochemical hot moments may arise. Beginning with the water itself, forest canopies intercept and redistribute stormwater, creating localized drip points, under which throughfall inputs can be more than 10 times greater than open rain (figure 1d; Zimmermann et al. 2009). If several branches efficiently capture and drain stormwaters to the stem, rainwater inputs to near-stem soils can be more than 100 times greater (Herwitz 1986). Downed coarse woody debris has also been found to collect and redirect rainwater to a concentrated area (Remsburg and Turner 2006). Canopy-draining stormwaters flush substantial quantities—but which are highly unpredictable across space and time—of inorganic nutrients (Ponette-González et al. 2020) and dissolved organic matter (tree-DOM). Tree-DOM visibly colors these waters (figure 1e; Stubbins et al. 2020), carries more easily available organic carbon to forest floors than is exported via streams or stored within the ecosystem, and may be critical to forests’ net carbon storage and export (Ryan et al. 2021, Behnke et al. 2022). Such localized inputs of easily available carbon (and energy) can boost microbial activity in hotspots over a short time (hot moments; Kuzyakov and Blagodatskaya 2015). Canopy stormwaters also carry biota, including millions of metazoans per tree per year (Ptatscheck et al. 2018) and billions of fungal spores per hectare per year, including newly discovered fungal species (Magyar et al. 2021).

The belated study of many aqueous hotpots and hot moments is surprising because they are visible to the human eye (Schumacher 1864, Bundt et al. 2001), albeit potentially missed by soil moisture sensors or lysimeters (*sensu* a century of denial of preferential flow paths; Beven 2018). These often-overlooked fluxes are not only apparent; they are impactful. Where visually apparent nutrient-rich waters enter dry soils, they can guide observers to potential localized sites to (technologically) monitor the ensuing bursts of decomposition and mineralization that produce carbon dioxide and inorganic nitrogen (Jarvis et al. 2007, Kuzyakov and Blagodatskaya 2015). Such short-term water fluxes can release nutrients from microbes through lysis (Sokol et al. 2022) and, when enriched with dissolved nutrients and organic carbon, they can trigger microbial activities and mineral weathering in deep soil (Fang et al. 2023), which favors new clay mineral formation (under incongruent weathering) or priming of organic matter decomposition (under congruent weathering). Visibly localized water fluxes may also form anaerobic soil microsites, subsequently spurring microbes to switch to alternative terminal electron acceptors (Fan et al. 2020) and proliferating a diversity of microbial metabolisms, including denitrification, manganese and iron reduction, and methanogenesis in forest soils (Keiluweit et al. 2016). Forest soils that are typically sinks for methane can shift to become sources when wet and anoxic conditions favor methanogenesis (Wang and Bettany 1997, Martins et al. 2021). Temporal trends show that methane uptake by forest soils may decline with increasing precipitation (Ni and Groffman 2018). However, measurements—and, therefore, knowledge—of soil–atmosphere gas exchanges are often discontinuous and biased toward dry or steady state conditions (Scott et al. 1999, Ford et al. 2012). Although automated infrastructure for monitoring gas efflux exists, it is expensive, logistically challenging, and spatially limited (missing hotspots; Fassbinder et al. 2013).

Microbial activities associated with transient, storm-related niches are observable by scientists who persist through the rain (Burgin et al. 2011). Oil-like sheen and rust-color particles on some puddles can appear in forests (figure 1f), reflecting iron-oxidizing bacteria in microsites of elevated or altered nutrient cycles. Such fluctuations between ferrous (iron(II)) and ferric (iron(III)) oxidation states also yield insights into interconnected cycles of other elements and molecules, including sulfur, nitrogen, phosphorus, biominerals, other metal or metalloid transformations (Li et al. 2012), organic carbon turnover (Hall and Silver 2013, Matus et al. 2019), lignin and cellulose decomposition (Merino et al. 2021a, 2021b, Du et al. 2020), and methane production (e.g., Dubinsky et al. 2010). Other visually observable cues of storm-related microbial activity can relate to elemental sulfur (white or pale yellow deposits; figure 1g) or green chloroplasts of photosynthesizing cyanobacteria and algae (figure 1h).

Smells can also cue humans into ephemeral microbial activities. Hydrogen sulfide gas from sulfate-reducing microbes smells like rotten eggs (Keiluweit et al. 2016). Although sulfate reduction and sulfide gas formation are anaerobic processes, well-drained and well-aerated soils can develop anoxic microsites (Keiluweit et al. 2018) and host sulfate reducing microbes who await favorable conditions (Peters and Conrad 1996). The smell of fresh rain is also microbially generated, mainly from terpenoids produced by *Streptomyces* bacteria and filamentous fungi (Yamada et al. 2015). Following their noses, scientists have been led to interesting discoveries. Becher and colleagues (2020) showed that these terpenoids attract springtails to aid in long-distance spore dispersal.

### Vegetation functions

Leaves, bark, and epiphytes are often wet. Their wetness can be estimated using sensors (Klemm et al. 2002) and energy balance models (Asdak et al. 1998), but these approaches may not reveal the incredible variation among leaf surfaces (figure 1i–1j). This variability in wetness has wide-reaching impacts—for example, by reducing or enhancing carbon uptake (Hanba et al. 2004, Misson et al. 2005, Aparecido et al. 2017), altering pathways of precipitation to the ground (Van Stan et al. 2011, Van Stan and Allen 2020), providing opportunities for leaf or stem water uptake and rehydration (Mayr et al. 2014, Mason Earles et al. 2016, Berry et al.^ 2019^, ^2021^), and capturing substantial moisture in barks and deadwood (Floriancic et al. 2022). In addition to the wetness state, the rapidity of transitions from wet to dry canopy conditions may also be consequential. For example, a recent study of a Japanese cypress forest showed that carbon dioxide uptake in the first few hours following periods of leaf wetness was higher than during typical dry periods (Jiao et al. 2021). Rain not only wets leaves but also renders light more diffuse, which can boost photosynthesis (Berry and Goldsmith 2020). Leaf gas-exchange measurements are among the most common activities of plant ecologists (Kattge et al. 2020), but Berry and Goldsmith (2020) only found three studies that assessed effects of leaf wetting on this key interface between the biosphere and atmosphere and eight studies focused on effects of diffuse light, with most of those studies lacking realism because they were conducted indoors, away from the rain and clouds.

Wandering a rain-soaked forest reveals the multitude of ways plants take advantage of storm-induced flow pathways. Rainy visits to Lord Howe Island (Australia) led Biddick and colleagues (2018) to discover roots *aboveground* that harvest water from preferential flow paths through the plant's own gutter-like leaves and branch channels (figure 1k–1m). Mosses, lichens, and other nonvascular epiphytes adapted to anhydrobiosis are dependent on canopy storm-related hydration–dehydration cycles, such as stemflow or storage and evaporation of water within bark (Porada and Giordani 2021). Because epiphytes depend on atmospheric water sources (Gauslaa 2014), observation of the type, intensity, and dynamics of precipitation becomes crucial to understanding their ecophysiology and their effect on ecosystem function. In some forests, the color of cyanolichens changes as they saturate with the storm, from white to green (because of the chlorophyll)—a color change that signals other biophysical changes, including fixation of atmospheric nitrogen, variation in albedo, and therefore a change of surface temperature (Aartsma et al. 2020). Stormwaters often exceed the water storage capacity of epiphytic vegetation, leading to overflow (Mendieta-Leiva et al. 2020) and nutrient leaching from the canopy (Coxson 1991, Van Stan and Pypker 2015). Following these stormwater and nutrient pulses, dry landscapes transform in ways that may unveil avenues toward the discovery of new life and processes.

### Animal behavior

Our umbrella perspective may conceal or misinterpret important animal behaviors and animal–environment interactions. For example, koalas were often described as not needing to drink, because they were rarely observed doing so. Opportunistic observations during storms revealed that koalas drink stemflow (figure 1n; Mella et al. 2020). Because koalas spend most of their time in trees and because storms make it hard to look upward, the natural drinking behavior of koalas was overlooked because scientists designed dry and comfortable observation methods. Improved understanding of koalas’ physiological need for free water has consequences for their conservation and habitat management. Maned sloths (*Bradypus torquatus*) share a similar story: Ecologists caught in a storm observed a sloth drinking from a branchflow path for the first time (de Albuquerque et al. 2021). Because this behavior had never been recorded before, it was previously assumed that sloths did not spontaneously ingest water.

Insect behaviors have also been observed to change during storms. Maschwitz and Moog (2000) reported that an ant colony prevented their bamboo nest from flooding by communally drinking stormwaters, then urinating in an area that would drain away from the nest. Rapid changes in humidity and air pressure can influence insect behavior (Wellington 1946), but these effects have primarily been studied during the dry periods between storms (Enjin 2017). Those studies that have reported observations regarding the effects of humidity on insect behavior before, during, and after storms have progressed theory. Approaching storms can increase foraging time for a honeybee species, *Apis mellifera* (He et al. 2016), and can reduce mating activities in three taxonomically unrelated insect species (Pellegrino et al. 2013). Immediately after storms, insect foraging behavior increases because the higher humidity reduces desiccation risk, and the stormwaters can uncover resources (Gordon et al. 2013). Therefore, our future presence in the storm could help uncover disregarded or overlooked aspects regarding how animals shelter, feed, hydrate, and die.

### Earth and planetary surface processes

Forests’ redistribution of stormwaters may influence sediment routing through watersheds, imparting biosignatures to underlying soils and sediments that are useful to reconstruct the distribution of forests through deep time. Therefore, scientist observations of and experiences in stormy forests today support efforts to understand Earth's geologic history and modern interactions within and between terrestrial and aquatic systems. For example, by the time storm events mobilize sediment along hillslopes and stream channels, the hydrologic information is already modified by the watershed effects that include the forests’ interception, capture, and routing of water to or through soils. Integrated over that forest's lifetime, which may be thousands to millions of years, precipitation partitioning by vegetation is one of innumerable sedimentary processes that must be considered when reconstructing important components of Earth history from the sedimentary record (e.g., paleoclimate, sea-level change, and tectonics; Jerolmack and Paola 2010). When canopies discharge intercepted water through drip points or stemflow, this can localize hydrologic, geomorphic, and sedimentary processes. Therefore, observations of canopy stormwater routing may inspire novel hypotheses regarding these waters’ capability to produce biosignatures (i.e., any morphological, chemical, or isotopic traces from an organism). Known forest biosignatures include precipitation of cements (possibly microbially aided; Perry et al. 2007) and rhizoliths (Gocke et al. 2011) or the opposite, the formation of dissolution features (Lipar et al. 2021). Finally, geomorphologists visiting landscapes during storms may open creative avenues for interpreting landscape features on other planets. The use of Earth-based analogs to explain geomorphological processes on other planetary bodies is a well-established method (Dypvik et al. 2021, Conway 2022). For example, comparison of sediment routing by storms through watersheds with forest canopies versus bare-Earth watersheds and its eventual deposition remains an unexplored space that could yield reasonable criteria for identifying forest biosignatures on planetary bodies.

### The umbrella perspective affects Earth system models

Earth system models (ESMs) contain many dry concepts that are applied to intrinsically wet conditions and systems, from the tops of trees to the ground surface and through soils. For example, the amount of rainwater retained in tree canopies is often estimated in ESMs theoretically (as 0.1–0.2 millimeters of storage per leaf area index) and is, in a sense, a dry equation that estimates low canopy water storage capacities, 0.1–2.0 millimeters (Klamerus-Iwan et al. 2020). The representation of precipitation-intercepting vegetation structure itself by ESMs is challenging, especially regarding disturbance effects (Fisher et al.^ 2018^, Fisher and Koven 2020) and nonleaf components (Porada et al. 2018, Van Stan et al. 2021). Wet scientists have observed additional water storage in nonleaf components, such as epiphytes (Zotz et al. 2020, Porada et al. 2018), water-filled tree holes (Magyar et al. 2021), and bark structures (Klamerus-Iwan et al. 2020) that collectively result in storage capacities exceeding the dry equation estimates by many times in many regions (Porada et al. 2018). A consequence of underestimating canopy water storage and, particularly, evaporation from wet leaf surfaces and epiphytes is a large potential bias in surface temperature simulated by ESMs (up to –0.6 Kelvin globally; Davies-Barnard et al. 2014).

Some ESM process representations of water storage by canopies are not completely dry but rely on few local observations collected by wet scientists. However, these damp model representations may rely on too few, too limited, or too localized wet observations to saturate theory with a robust set of observational support across systems. For example, Lundquist and colleagues (2021) found that ESM representations of forest snow interception are based on data collected from just two storms in Idaho (in the United States). It is perhaps no wonder, then, that these damp models have large uncertainties in their predictions of snowy, forested regions’ hydrologic response to climate change (Lundquist et al. 2021).

Stormwater stored on leaves influences canopy conductance, affecting ESM estimates of carbon fixation and transpiration. However, ESMs either deploy literally dry equations, ignoring the effect of canopy wetness on conductance, or halt gas exchange when leaves are wet (e.g., Bonan et al.^ 2018^). Evidence from wet leaf observations, including direct measurements of wet leaf photosynthesis (Aparecido et al. 2017, Berry and Goldsmith 2020), demonstrate that these dry modeling approaches and assumptions are incorrect. Scientists’ observations and shower thoughts on this topic are crucial, because plant gas exchange methods lag behind advances in understanding canopy wetting patterns (Binks et al.^ 2022^). For epiphytes, the relevance of stormwater storage has been questioned as they were assumed to be regularly close to saturation. Field observations and process-based modeling have shown, however, that saturation only occurs approximately 20% of the time (Hargis et al.^ 2019^), supporting epiphytes’ inclusion in ESMs (making the equations wetter). Although process-based vegetation models (e.g., Porada et al. 2018) can represent fast water pool dynamics during storms, they still lack key processes at the intersection of ecophysiology and biogeochemistry that regularly occur during these hot moments. For example, respiration pulses in nonvascular epiphytes on rewetting can affect their long-term carbon balance (Brown et al. 1983), and storm rewetting can lead to nutrient release pulses in tropical nonvascular epiphytes (Coxson 1991) affecting organisms’ nutrient budget and whole-forest cycling (Clark et al. 1998). Epiphytes remain neglected in major ESMs, making these models dry in both parameterization and at the process level.

Given that storm–ecosystem interactions contribute to landscape evolution (Lyell 1834, Collins et al. 2004), ESMs’ dry process representations can influence our understanding of the past. A key example of this is the stream power law, which relates local channel bed incision to the area and slope of the contributing watershed. In application, this relationship allows the simulation of landscape evolution through deep time with minimal computational cost. Although an intrinsically dry geometric scaling law, additional realism of our wet world was instilled by an adaptation to consider bedrock weathering rates as a function of precipitation gradients across watersheds (Murphy et al. 2016) accelerated by carbon and nutrient flushes (Fang et al. 2023). Below the surface, rapid bypass flow through soil macropores occurring during storms can represent 1%–70% of subsurface water movement, often influencing water and solute exports from ecosystems (Radolinski et al. 2022). However, ESMs rely on a damp representation of subsurface flow: the Buckingham–Richards equation, a uniform flow equation derived from rigorous (over)controlled lab conditions (Beven 2018, Swenson et al. 2019). Although ESMs will always be (necessarily) incomplete, the observations and shower thoughts of wet scientists will be useful in pointing us toward areas and conditions where sampling and technological monitoring may best help hydrate established, simple dry modeling approaches.

## Soaking in ideas: Making environmental education more immersive

Fostering a solid foundation for future work requires integrating the innovative thinking inspired by shower thoughts in environmental education. Educational experiences that encourage immersion in natural environments during storms may be key to leveraging technology, uniting human observation, modeling, and remote sensing to advance knowledge across generations. Immersive learning in environmental education should expose students (and researchers at all career stages) to the broadest possible range of ecosystem conditions. Imagination and technology are powerful tools, but perhaps it will be the personal presence of educators and students that inspires creative solutions to current limitations in ESM representations of stormy phenomena and the ecosystem structures and disturbances affecting them. Importantly, student experiences in storms can be both targeted at known emergent phenomena (such as the examples provided) and given the freedom to explore and discover. When student experiences become too narrowly targeted by educators, we risk treating students like technological sensors and, thereby, risk constraining their personal ability to muse over something that may deepen their wonder and appreciation of nature and, ultimately, a broader community's understanding of and connection to the ecosystems in which they live.

On this subject, philosopher Friedrich Nietzsche once wrote, “beware of interrupting a student's naive, confident, and, as it were, immediate and personal relationship with nature! The woods, the rocks, the winds, the vulture, the flowers, the butterfly, the meads, the mountain slopes must all speak to them in their own language; in them, one must, as it were, come to know oneself again in countless reflections and images, in a variegated round of changing visions, and in this way one will unconsciously and gradually feel the metaphysical unity of all things in the great image of nature and, at the same time, tranquilize one's soul in the contemplation of her eternal endurance and necessity” (Nietzsche 1872). Encouraging mindfulness and direct observations of phenomena during storms across disciplines may lead to discoveries that bridge gaps in knowledge and foster a more holistic understanding of ecosystems. Therefore, rather than charting specific future directions, we invite students, educators, and researchers to engage with nature during storms, embracing the potential for new insights and deeper connections to the world around them. After all, who knows what another's eyes, nose, skin, or ears may discover and how it may empower researchers to advance ecosystem science and inform sustainable management practices, all while nurturing their connection to the natural world?

## Let's close the umbrella!

Natural scientists seem increasingly content to stay dry and rely on remote sensors and samplers, models, and virtual experiments to understand natural systems. Consequently, we can miss important stormy phenomena, imaginative inspirations, and opportunities to build intuition—all of which are critical to scientific progress. The limitations of a dry umbrella perspective will likely become more costly as global average precipitation continues to rise and precipitation increases in frequency and intensity in many regions of the world. For example, local-, regional-, and global-scale studies of forests have revealed somewhat surprising negative anomalies in plant productivity and photosynthetic activity that correspond with *wetter* conditions (Hubbart et al. 2016, Li et al. 2022), but these forest responses to precipitation remain mysterious because they are understudied. Therefore, like others, we call for expanded study of the impacts of anomalously wet events and seasons to parallel the one-sided proliferation of drought studies. The combination of human experiences in the storm, our shower thoughts, with technological tools arguably produce the best odds for scientific advancement. Although we focused on forests, the shade of our sheltered, umbrella perspective likely darkens our understanding of all natural and human systems. Storms produce even more important event-driven processes in semiarid ecosystems, whose response is not buffered by the forest canopy. Our call, therefore, is for those who study natural and socioecological systems to enter the storm (with caution, of course) to collect human observations that complement other methods. We also challenge funding agencies, many of which have tilted support toward remote sensing, to explicitly support activities that place researchers in the storm.

## Author Biography

J.T. Van Stan (j.vanstan@csuohio.edu) and K.E. Mueller are affiliated with the Department of Biological, Geological, and Environmental Sciences at Cleveland State University, in Cleveland, Ohio, in the United States. S.T. Allen is affiliated with the Department of Natural Resources and Environmental Science at the University of Nevada-Reno, in Reno, Nevada, in the United States. D.P. Aubrey is affiliated with Savannah River Ecology Lab and with the Warnell School of Forestry at the University of Georgia, in Athens, Georgia, in the United States. Z.C. Berry is affiliated with the Department of Biology at Wake Forest University, in Winston-Salem, North Carolina, in the United States. M. Biddick is affiliated with the Terrestrial Ecology Research Group at the Technical University of Munich, in Freising, Germany. A.M.J. Coenders-Gerrits is affiliated with the Department of Water Management at the Delft University of Technology, in Delft, in the Netherlands. P. Giordani is affiliated with the Dipartimento di Farmacia at the University of Genoa, in Genoa, Italy. S.G. Gotsch is affiliated with the Department of Forestry and Natural Resources at the University of Kentucky, in Lexington, Kentucky, in the United States. E.D. Gutmann is affiliated with the Research Applications Laboratory, at the National Center for Atmospheric Research, in Boulder, Colorado, in the United States. Y. Kuzyakov is affiliated with the Department of Soil Science of Temperate Systems, Agricultural Soil Science, at Georg-August-Universität, in Göttingen, Germany, and with the Peoples Friendship University of Russia, in Moscow, Russia. D. Magyar is affiliated with the National Public Health Center, in Budapest, Hungary. V.S.A. Mella is affiliated with the Sydney School of Veterinary Science, at the University of Sydney, in Sydney, New South Wales, Australia. A.G. Ponette-González is affiliated with the Department of City and Metropolitan Planning and with the Natural History Museum of Utah at the University of Utah, in Salt Lake City, Utah, in the United States. P. Porada is affiliated with the Department of Biology at Universität Hamburg, in Hamburg, Germany. C.E. Rosenfeld is affiliated with the Department of Minerals and Earth Sciences at the Carnegie Museum of Natural History, in Pittsburgh, Pennsylvania, in the United States. J. Simmons is affiliated with the Department of Philosophy and Religious Studies at Georgia Southern University, in Statesboro, Georgia, in the United States. K.R. Sridhar is affiliated with the Department of Biosciences at Mangalore University, in Konaje, Mangaluru, Karnataka, India. A. Stubbins is affiliated with the Departments of Marine and Environmental Science, Civil and Environmental Engineering, and Chemistry and Chemical Biology at Northeastern University, in Boston, Massachusetts, in the United States. T. Swanson is an independent scholar in Savannah, Georgia, in the United States.
